# Increased non-typhoidal *Salmonella* hospitalizations in transfusion-naïve thalassemia children: a nationwide population-based cohort study

**DOI:** 10.1038/s41390-021-01602-7

**Published:** 2021-06-19

**Authors:** Jiunn-Ming Sheen, Fang-Ju Lin, Yao-Hsu Yang, Kuang-Che Kuo

**Affiliations:** 1 Department of Pediatrics, Kaohsiung Chang Gung Memorial Hospital and Chang Gung University College of Medicine, Kaohsiung, Taiwan; 2 Department of Pediatrics, Chiayi Chang Gung Memorial Hospital and Chang Gung University College of Medicine, Chiayi, Taiwan; 3 Department of Traditional Chinese Medicine, Chang Gung Memorial Hospital, Chiayi, Taiwan; 4 Institute of Occupational Medicine and Industrial Hygiene, National Taiwan University College of Public Health, Taipei, Taiwan

## Abstract

**Introduction:**

Although non-typhoidal *Salmonella* (NTS) infection usually causes self-limited enterocolitis, several risk factors have been found to predispose individuals to more severe NTS infections. However, few studies have discussed the association between NTS infection and pediatric thalassemia populations.

**Material and methods:**

A nationwide population-based retrospective cohort study was conducted using medical records of the selected children from the Taiwan National Health Insurance Research Database. Immunocompromised individuals or patients with a history of transfusion or splenectomy were excluded. One thalassemia patient was matched with four non-thalassemia patients based on their year of birth, sex, and urbanization level.

**Results:**

In this cohort, 912 patients with thalassemia and 3648 comparison cohort were analyzed. The mean age of NTS hospitalization was 2.0 ± 1.4 in thalassemia cohort and 2.6 ± 2.4 in non-thalassemia cohort. Transfusion-naïve thalassemia children were proved to have a higher rate of NTS hospitalization (6.90 vs 4.11 per 1000 person-year; *p* = 0.0004) than the non-thalassemia cohort, with an adjusted hazard ratio (HR) of 1.68 (95% confidence interval [CI] = 1.26–2.24).

**Conclusion:**

Our research shows that transfusion-naïve thalassemia is associated with an increased risk of NTS hospitalization. Further prospective study comparing the incidence and severity of NTS infection among children with and without thalassemia is needed.

**Impact:**

- Pediatric transfusion-naïve thalassemia patients have an 1.68-fold increased risk for hospitalization due to non-typhoidal *Salmonella* (NTS) infection.
- This is the first nationwide population-based cohort study based on an extremely large database that shows pediatric transfusion-naïve thalassemia patients have an increased risk for NTS hospitalizations.
- Besides the previously known risk factors such as extremes of age, sickle cell disease, or immunosuppressing conditions, clinicians must also take thalassemia as a possible risk factor for more severe NTS disease.

## Introduction

Non-typhoidal *Salmonella* (NTS) infection has been and continues to be a serious health issue worldwide. According to the Global Burden of Diseases, Injuries, and Risk Factors Study (GBD) 2017, there were an estimated 95.1 million cases,^1^ and 535,000 invasive NTS infections globally in 2017.^2^ NTS are usually caused by contacting animals, drinking contaminated water, or consuming contaminated food products, especially poultry and eggs.^3–7^ Despite the significant improvement in public health in Taiwan, NTS infection has remained as the most common cause of bacterial enterocolitis among children requiring hospitalization.^8^ Although NTS infection usually causes self-limited enterocolitis, risk factors such as extremes of age, immunosuppressing conditions, and hemoglobinopathies predispose individuals to invasive NTS disease.^9–12^ In developed countries, NTS infection frequently shows as a primary bacteremia in adults, while it often remains secondary to gastroenteritis in children.^13–15^ The reported incidence of bacteremia among children with NTS gastroenteritis varies widely, from 3 to 11.4%,^16–19^ depending on geographical region, age, and underlying disease(s) of the individual. Although most NTS bacteremia in immunocompetent children is readily manageable with adequate antibiotic treatments, the economic burden is much heavier compared to self-limited NTS enterocolitis.^14,20^ Furthermore, while rarely seen in previously healthy immunocompetent children, the focal suppurative disease, which causes the prolonged length of hospital stay and may require major surgical intervention,^21,22^ does occur in some cases. Compared to adults, children with NTS bacteremia are less often to be identified with predisposing diseases.^13^ In children, sickle cell disease is the most studied hemoglobinopathy in relation to invasive NTS infection.^12,23^ Sickle cell disease is not prevalent in Taiwan; nevertheless, carriers for α‐ or β‐thalassemia in the country takes up 6.2% of our 23 million population.^24–26^ However, studies on the association between NTS infection and thalassemia in children are insufficient. In this study, we aim to investigate the relationship between transfusion-naïve thalassemia and NTS infection.

## Methods

### Data source

The Taiwan National Health Insurance (NHI) program is a single-payer system instituted on 1 March 1995, which, according to the Bureau of the National Health Insurance (BNHI), covers >99% of the 23 million citizens of Taiwan. The BNHI has authorized the National Health Research Institutes (NHRI) to create the National Health Insurance Research Database (NHIRD) for medical researches. This database contains administrative and health claims data collected through the NHI program, including complete information on inpatient care, ambulatory care, dental care, and prescription drugs. It provides researchers with scrambled identification numbers associated with relevant claims information, including records on patients' sex, date of birth, registry of medical services, and medication prescriptions. In this study, we used the Longitudinal Health Insurance Database 2010 (LHID2010), which is a subset of the NHIRD comprising patients’ data from 1996 to 2013. The LHID2010 includes data of 1,000,000 beneficiaries randomly sampled from the original NHIRD. Diseases were defined according to the International Classification of Diseases, Ninth Revision, Clinical Modification (ICD-9-CM).^27^ This study was approved by the Institutional Review Board of Kaohsiung Chang Gung Memorial Hospital (Permit No CGMF- 20181208B0C501), Taiwan.

### Sampled patients

Patients who were born between 1997 and 2010, and diagnosed with thalassemia, which is defined as the ICD-9-CM code 282.4 being documented twice or more in the ambulatory claims or if it was ever recorded in an inpatient diagnosis, were included in our study cohort. As the data set comprises patients’ data from 1997 to 2013, the recruited cohort were either children or adolescents. We excluded patients who had HIV infection (ICD-9: 042), congenital immunodeficiency (ICD-9: 279.X), asplenia (ICD-9: 759.0), or those who received total (ICD-9: 41.5) or partial splenectomy (ICD-9: 41.43). In addition, we excluded cases with a history of transfusion (ICD-9:990 or treatment code 94005C, 94005c).

**Fig. 1:**
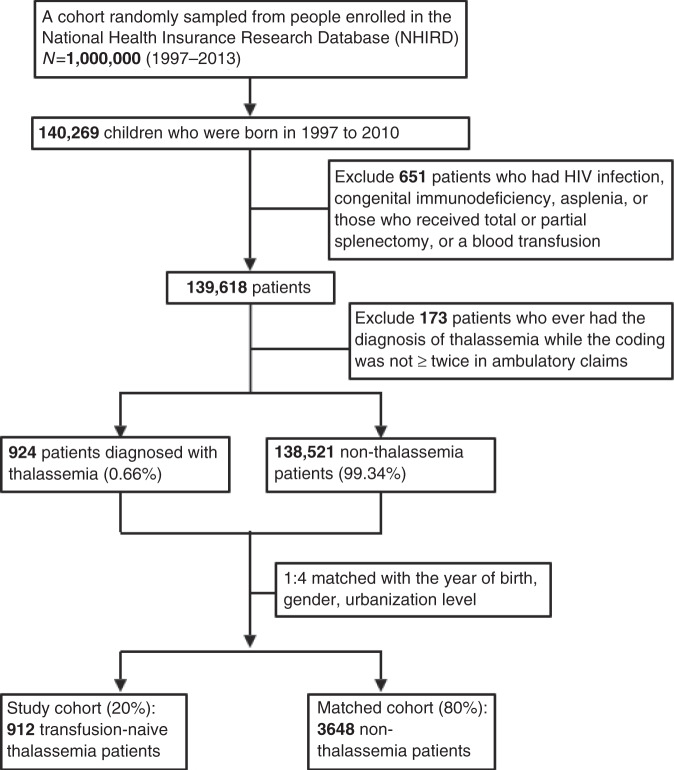
Flowchart of enrollment of the study patients. HIV human immunodeficiency virus.

The flowchart of the patient enrollment process for the study group is presented in Fig. 1. Every single thalassemia patient was matched with four non-thalassemia individuals (1:4 matching) based on their year of birth, sex, and the urbanization level, with level 1 referring to the most urbanized cities and level 4 to rural villages.^28^ Comorbidities were also investigated and they were counted if the ICD-9 codes were documented for three times or more in the ambulatory claims, or if they were ever recorded in inpatient diagnosis before hospitalization due to non-typhoidal *Salmonella*. These comorbidities include diabetes mellitus (ICD-9: 250), biliary atresia (ICD-9: 751.61), nephrotic syndrome (ICD-9: 581), chronic kidney disease (ICD-9: 585, 586, 403, 404), and renal dialysis(ICD-9: V45.1, V56, ICD-op: 3995, Health Insurance treatment order: 58802, 58029, 58001, 58027), autoimmune disease including systemic lupus erythematosus (ICD-9: 710.0), rheumatoid arthritis (ICD-9: 714.0), juvenile idiopathic arthritis (ICD-9: 714.2–714.3), psoriasis (ICD-9: 696.0, 696.1, 696.2, 696.8), and other miscellaneous autoimmune disease (ICD-9: 710.1, 710.2, 710.3, 725).

### Outcome

The patients in both thalassemia and non-thalassemia cohorts were tracked until they were hospitalized due to non-typhoidal *Salmonella* gastroenteritis (ICD-9: 0030), *Salmonella* bacteremia (ICD-9: 0031), *Salmonella* meningitis (ICD-9: 00321), *Salmonella* arthritis (ICD-9: 00323), *Salmonella* osteomyelitis (ICD-9: 0039), other specified *Salmonella* infections (ICD-9: 0038), or until death occurred, or to the end of 2013.

### Statistical analysis

The distributions of categorical demographics and clinical characteristics between the thalassemia and non-thalassemia cohorts were compared using the chi-square test. The incidence rate ratios (IRRs) and 95% confidence intervals (CIs) were estimated using Poisson regression. Univariate and multivariate Cox proportional hazard regression models were used to estimate the hazard ratios (HRs) and 95% CIs for NTS hospitalizations. To demonstrate consistency, we also performed subgroup analyses to determine whether the results remained significant in different sex, birth year, and urbanization level groups. Kaplan–Meier method was used to calculate the cumulative incidence rates of NTS hospitalization in both cohorts, and the log-rank test was used to analyze the differences between the curves. The proportionality test was performed to ensure that the essential assumption of the Cox regression models was not violated.^29^ All statistical analyses were performed using SAS software, Version 9.3 (SAS Institute, Cary, NC). A *P* value <0.05 in two-tailed tests was considered statistically significant.

## Result

A total of 912 pediatric patients with transfusion-naïve thalassemia and a matched cohort of 3648 non-thalassemia patients were analyzed. Of the 912 transfusion-naïve thalassemia pediatric patients, 379 (41.6%) were female. Three hundred and forty-seven patients (38.1%) were born between 1997 and 2000, 307 (33.7%) between 2001 and 2005, and 258 (28.3%) between 2006 and 2010. Two hundred and sixty-seven (29.3%) transfusion-naïve thalassemia patients resided in communities of the highest urbanization level, 444 (48.7%) in the second highest, 141 (15.5%) in the third, and 60 (6.6%) lived in rural villages. There were no significant differences in age, sex, or urbanization level between two groups. Few comorbidities have been documented in this study (Table 1).

**Table 1:** Characteristics of thalassemia patients and the matched controls.

Variables	Thalassemia	Non-thalassemia	*P* value
	*N* = 912	*N* = 3648	
Gender, *n* (%)			
Female	379 (41.6)	1516 (41.6)	>0.999
Male	533 (58.4)	2132 (58.4)	
Birth year, *n* (%)			
1997–2000	347 (38.1)	1388 (38.1)	>0.999
2001–2005	307 (33.7)	1228 (33.7)	
2006–2010	258 (28.3)	1032 (28.3)	
Urbanlization level, *n* (%)			
1 (cities), *n* (%)	267 (29.3)	1068 (29.3)	>0.999
2, *n* (%)	444 (48.7)	1776 (48.7)	
3, *n* (%)	141 (15.5)	564 (15.5)	
4 (villages), *n* (%)	60 (6.6)	240 (6.6)	
Follow-up periods, years	10.33 ± 4.50	10.54 ± 4.26	
NTS infection, *n* (%)	65 (7.1)	158 (4.3)	0.0008
Diabetes mellitus, *n* (%)	3 (0.3)	1 (0)	
SLE, *n* (%)	1 (0.1)	1 (0)	
RA, *n* (%)	1 (0.1)	2 (0.1)	
JIA, *n* (%)	2 (0.2)	1 (0)	
Psoriasis, *n* (%)	0(0)	2 (0.1)	
Other autoimmune disease, *n* (%)	2 (0.2)	2 (0.1)	
Biliary atresia, %	0 (0)	1 (0)	
Nephrotic syndrome, *n* (%)	4 (0.4)	1 (0)	
Chronic kidney disease, *n* (%)	1 (0.1)	0 (0)	
Renal dialysis, *n* (%)	0 (0.0)	0 (0.0)	

*SLE* systematic lupus eruthematosus, *RA* rheumatoid arthritis, *JIA* juvenile idiopathic arthritis, *NTS* non-typhoidal *Salmonella*.

**Fig. 2:**
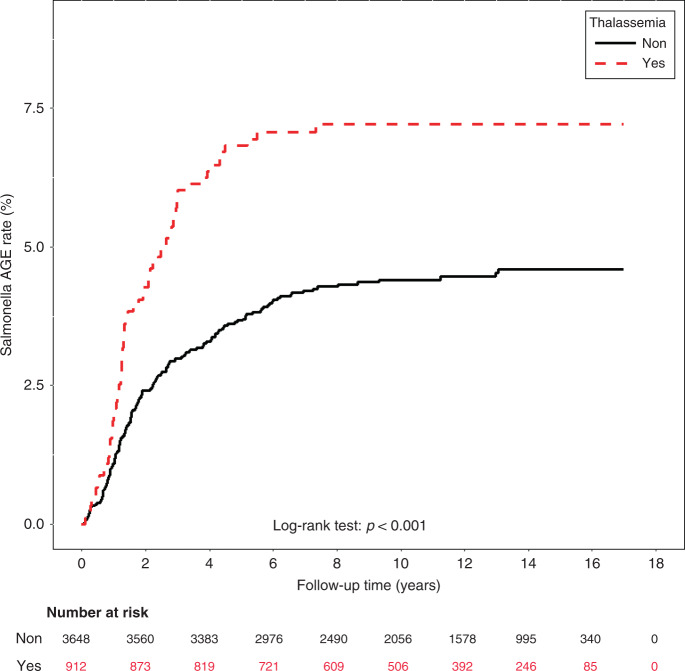
Cumulative incidence comparison of hospitalization due to non-typhoidal *Salmonella* infection. Dashed line indicates patients with thalassemia and solid line refers to individuals without thalassemia.

The mean follow-up years were 10.33 ± 4.50 and 10.54 ± 4.26 for the thalassemia cohort and the non-thalassemia cohort, respectively (Table 1). Sixty-five transfusion-naïve thalassemia and 158 non-thalassemia cases (cumulative incidence 7.1% and 4.3% respectively, *p* = 0.0008) were hospitalized due to NTS infection in this cohort (Table 1). The mean age of NTS hospitalization was 2.0 ± 1.4 in thalassemia cohort and 2.6 ± 2.4 in non-thalassemia cohort (*p* = 0.001). Figure 2 shows that the cumulative incidence of hospitalization due to NTS infection was significantly higher in the transfusion-naïve thalassemia cohort than in the non-thalassemia cohort (log-rank test *p* < 0.001).

After birth year, sex, and urbanization level have been adjusted, the overall incidence of hospitalization due to NTS infection was 1.68-fold higher (95% CI: 1.26–2.24) in transfusion-naïve thalassemia than in non-thalassemia cohort (6.90 and 4.11 per 1,000 person-years, respectively). Subgroup analysis shows that the effect of thalassemia remained significant for both sexes, for those born between 2006 and 2010, and for patients with the second highest urbanization level (Table 2).

**Table 2:** Incidence of hospitalization due to non-typhoidal *Salmonella* by gender, birth year, and urbanization levels and Cox model measured hazard ratios for patients with transfusion-naïve thalassemia compared to non-thalassemia cohort.

	Thalassemia (*N* = 912)				Non-thalassemia (*N* = 3648)				Thalassemia to non-thalassemia			
	Event	*N*	PY	Rate^a^	Event	*N*	PY	Rate^a^	Crude HR (95% CI)	*P* value	Adjusted HR ^b^ (95% CI)	*P* value
Overall	65	912	9421.7	6.90	158	3648	38449.0	4.11	1.67 (1.25–2.24)	0.0005	1.68 (1.26–2.24)	0.0004
Gender												
Female	27	379	3945.3	6.84	67	1516	15958.7	4.20	1.63 (1.05–2.55)	0.0313	1.63 (1.04–2.55)	0.0321
Male	38	533	5746.5	6.61	91	2132	22490.2	4.05	1.70 (1.17–2.49)	0.0058	1.63 (1.12–2.39)	0.011
Birth year												
1997–2000	22	347	4946.8	4.45	69	1388	20039.5	3.44	1.29 (0.80–2.08)	0.3019	1.29 (0.80–2.09)	0.2959
2001–2005	23	307	3069.6	7.49	63	1228	12534.8	5.03	1.49 (0.92–2.40)	0.1036	1.49 (0.92–2.40)	0.1012
2006–2010	20	258	1405.4	14.23	26	1032	5874.7	4.43	3.17 (1.77–5.67)	0.0001	3.22 (1.80–5.76)	<0.0001
Urbanization level												
1 (cities)	12	267	2744.5	4.37	43	1068	10931.2	3.93	1.12 (0.59–2.12)	0.7286	1.11 (0.59–2.11)	0.7461
2	40	444	4518.4	8.85	90	1776	18675.9	4.82	1.83 (1.26–2.65)	0.0015	1.84 (1.27–2.67)	0.0014
3	10	141	1522.4	6.57	21	564	6275.6	3.35	1.94 (0.92–4.13)	0.084	1.96 (0.92–4.17)	0.0792
4 (villages)	3	60	636.4	4.71	4	240	2566.3	1.56	3.03 (0.68–13.54)	0.1465	3.02 (0.68–13.51)	0.1473

*CI* confidence interval, *HR* hazard ratio, *PY* person-years.

^a^Incidence rate per 1000 person-years.

^b^Multivariable analysis including age, gender, and urbanization level.

## Discussion

To the best of our knowledge, this is the first nationwide population-based cohort study that draws upon an extremely large database featuring pediatric transfusion-naïve thalassemia patients have an 1.68-fold increased risk for hospitalization due to NTS disease. The patients’ birth year has been adjusted, and patients with NTS hospitalization were younger in thalassemia cohort than in non-thalassemia cohort, suggesting thalassemia patients have an increased risk for hospitalization at a younger age. Infections are major complications in patients with thalassemia, especially in those with thalassemia major.^30^ Iron overload, splenectomy, transfusion-related infection, impaired of both innate and adaptive immunity were the proposed mechanism for increased infection in thalassemia patients.^31^ Since 1993, Taiwan started a nationwide thalassemia screening upon pregnant women. There were an estimated 91% reduction in the incidence of thalassemia major.^32^ In this study, although we could not precisely identify different types and levels of severity of thalassemia owing to the limitation of ICD-9 coding system, we excluded those who had experienced blood transfusion or had received splenectomy. The remaining cases with thalassemia were therefore considered as the less severe ones. There were few studies discussing the relationship between thalassemia of lesser severity, e.g., thalassemia minor or thalassemia traits, and *Salmonella* infection. A previous study from Israel revealed that the likelihood of NTS bacteremia versus salmonellosis has an inverse association with hemoglobin level in adult,^33^ while the etiology of anemia was not determined in the study. Lee et al.^34^ analyzed 244 immunocompetent pediatric inpatients with non-typhoidal salmonellosis in a tertiary medical center in northern Taiwan from 2010 to 2018, and found that cases with NTS bacteremia were associated with a significant higher rate of anemia due to either thalassemia trait or prolonged disease course than the non-bacteremia patients, suggesting thalassemia trait might be a risk factor for more severe NTS infection. Non-typhoidal salmonellosis is usually self-limited and may not require hospitalization or antibiotic treatment. Although there were only five cases of invasive NTS infection in our study (two cases from thalassemia cohort, three from non-thalassemia cohort, data not shown), we found that the incidence of hospitalization due to NTS infection has increased in patients with thalassemia. Possible confounders toward NTS hospitalizations such as sanitation of their living environment and medical accessibility were adjusted, suggesting that transfusion-naïve thalassemia is a risk factor for more severe NTS infection requiring hospitalization.

The effect of thalassemia on NTS hospitalization remained robust in the subgroup analysis. Patients with thalassemia had an increased risk for NTS hospitalization in different birth cohorts and of different urbanization levels. However, the differences were only statistically significant for those born between 2006 and 2010, and for patients with the second highest urbanization level. The patients who were born between 2006 and 2010 were tracked for 3–7 years until the end of 2013, meaning these patients were equal to or less than 7 years of age. This significant finding in this birth cohort indicates that thalassemia has the most effect on NTS hospitalizations for patients at a younger age. Besides, of all cases who had been hospitalized due to NTS disease, more than 90% of the children were younger than 5 years old (data not shown). This pattern resonates with previous findings in other studies that NTS disease occurs at a younger age.^14,19^ The development of NTS disease depends on the result of the interaction between the pathogen and host immune responses. The innate immune system provides critical first-line defense against NTS and may be the determining factor in whether the infection is subclinical or more invasive.^35,36^ Immature innate immune system featuring impaired chemotactic activity of the neutrophils and altered phagocytosis of the monocytes has been identified among young children.^37^ Besides, evidence shows that even non-transfusion-dependent thalassemia predisposes individuals to defective innate immunity response.^31,38^ Taken together, this may explain why young children with thalassemia were even more susceptible to NTS infection. On a separate note, it is unclear as to why the differences were only statistically significant for patients with the second highest urbanization level. However, it is noteworthy that the majority of the patients in this cohort were with the second highest urbanization level, and this might explain the reason why the difference in this subgroup is significant.

There are however some limitations in this study. Firstly, this is a nationwide population-based cohort study using ICD-9 code to define disease. Without microbiologic data, some NTS infection might only be coded as infectious colitis in the database. Therefore, there might be an underestimation of NTS disease burden in this study. Secondly, the ICD-9 CM coding cannot identify the type or severity of thalassemia. Nonetheless, we excluded all thalassemia major patients by blood transfusion, so the remaining cases with thalassemia were therefore considered as the less severe ones. Thirdly, the patients' thalassemia diagnosis, just by virtue of having a comorbid condition, could play a crucial factor in determining their need to hospitalize. However, as mentioned above, the cases with thalassemia were the less severe ones in this cohort; thus, the effect might be of minor concern. Finally, the proportion of thalassemia patients in this cohort was 0.66%, which is far less than the estimated thalassemia carriers (6.2%) in Taiwan, suggesting that some asymptomatic carriers were not documented in the database. This indicates that there might have been some thalassemia patients in the comparison cohort, further noting that the NTS hospitalization rate in the comparison cohort may have been overestimated. In other words, the difference of NTS hospitalization rate between thalassemia and non-thalassemia cohort may be even bigger.

## Conclusion

Our study features the use of a nationwide population-based database that includes a large number of thalassemia cases. In this study, we found that underlying thalassemia is associated with an increased risk of NTS hospitalization after adjusting possible confounding factors. As NTS infection remains the most common cause of bacterial enterocolitis of children requiring hospitalization, clinicians should consider thalassemia as a risk factor for more severe NTS infections. Further prospective study comparing the incidence of NTS infection in children with and without thalassemia is needed.

## Supplementary information

2020 [MR] NTS thalassemia - explaining birth cohort for review
