# Correction: Increased non-typhoidal *Salmonella* hospitalizations in transfusion-naïve thalassemia children: a nationwide population-based cohort study

**DOI:** 10.1038/s41390-021-01662-9

**Published:** 2021-09-03

**Authors:** Jiunn-Ming Sheen, Fang-Ju Lin, Yao-Hsu Yang, Kuang-Che Kuo

**Affiliations:** 1 Department of Pediatrics, Kaohsiung Chang Gung Memorial Hospital and Chang Gung University College of Medicine, Kaohsiung, Taiwan; 2 Department of Pediatrics, Chiayi Chang Gung Memorial Hospital and Chang Gung University College of Medicine, Chiayi, Taiwan; 3 Department of Traditional Chinese Medicine, Chang Gung Memorial Hospital, Chiayi, Taiwan; 4 Institute of Occupational Medicine and Industrial Hygiene, National Taiwan University College of Public Health, Taipei, Taiwan

Correction to: *Pediatric Research* 10.1038/s41390-021-01602-7, published online 19 June 2021

In the original article, the order of authors was wrong and a footnote was missing. The original article has been corrected.

