# Supplementary figures and images for: Increased non-typhoidal Salmonella hospitalizations in transfusion-naïve thalassemia children: a nationwide population-based cohort study
Source: Pediatr Res. 2021 Jun 19;91(7):1858–63. doi: 10.1038/s41390-021-01602-7 (PMC9270223; doi:10.1038/s41390-021-01602-7)

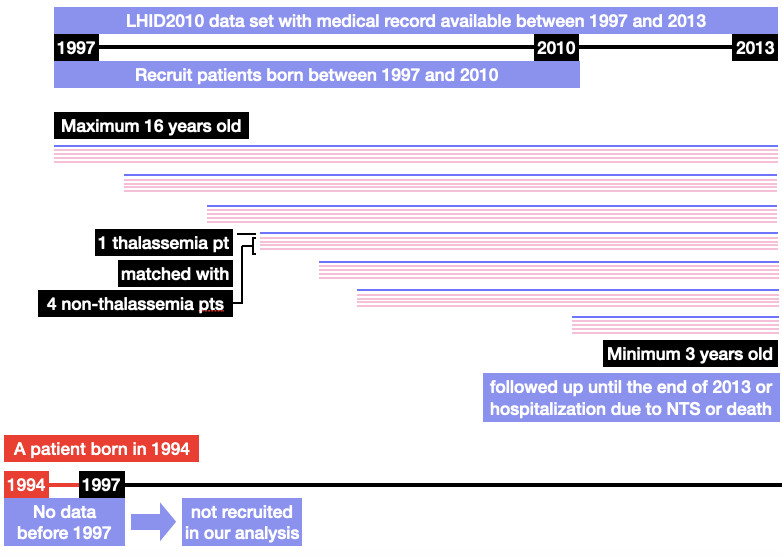

Supplement: Supplementary file 1 — 2020 [MR] NTS thalassemia - explaining birth cohort for review [file 41390_2021_1602_MOESM1_ESM.jpg]
